# Empagliflozin induces the transcriptional program for nutrient homeostasis in skeletal muscle in normal mice

**DOI:** 10.1038/s41598-023-45390-y

**Published:** 2023-10-21

**Authors:** Ryo Kawakami, Hiroki Matsui, Miki Matsui, Tatsuya Iso, Tomoyuki Yokoyama, Hideki Ishii, Masahiko Kurabayashi

**Affiliations:** 1 Department of Cardiovascular Medicine, Gunma University Graduate School of Medicine, 3-39-15 Showa-machi, Maebashi, Gunma 371-8511 Japan; 2 Department of Laboratory Sciences, Gunma University Graduate School of Health Sciences, Maebashi, Gunma Japan

**Keywords:** Cardiology, Medical research, Molecular medicine

## Abstract

Sodium-glucose cotransporter 2 inhibitors (SGLT2i) improve heart failure (HF) outcomes across a range of patient characteristics. A hypothesis that SGLT2i induce metabolic change similar to fasting has recently been proposed to explain their profound clinical benefits. However, it remains unclear whether SGLT2i primarily induce this change in physiological settings. Here, we demonstrate that empagliflozin administration under ad libitum feeding did not cause weight loss but did increase transcripts of the key nutrient sensors, AMP-activated protein kinase and nicotinamide phosphoribosyltransferase, and the master regulator of mitochondrial gene expression, PGC-1α, in quadriceps muscle in healthy mice. Expression of these genes correlated with that of PPARα and PPARδ target genes related to mitochondrial metabolism and oxidative stress response, and also correlated with serum ketone body β-hydroxybutyrate. These results were not observed in the heart. Collectively, this study revealed that empagliflozin activates transcriptional programs critical for sensing and adaptation to nutrient availability intrinsic to skeletal muscle rather than the heart even in normocaloric condition. As activation of PGC-1α is sufficient for metabolic switch from fatigable, glycolytic metabolism toward fatigue-resistant, oxidative mechanism in skeletal muscle myofibers, our findings may partly explain the improvement of exercise tolerance in patients with HF receiving empagliflozin.

## Introduction

Empagliflozin and dapagliflozin, two members of sodium-glucose cotransporter 2 inhibitors (SGLT2i) which promote urinary glucose loss, profoundly reduce the risk of hospitalization for heart failure (HF) across broad range of patient characteristics^1–3^. The physiological and biomolecular mechanisms responsible for this benefit are under intense investigation. One intriguing hypothesis that has recently emerged is that SGLT2i promote stress resistance and autophagic flux by upregulating nutrient-deprivation signaling in tissues including heart^4^. This mechanism has been proposed as a unifying hypothesis to explain the observed effects in randomized clinical trials and experimental evidence derived from rodent model of diabetes and HF, which are invariably associated with dysregulated energy homeostasis.

The nutrient-deprivation signaling by SGLT2i may be relevant to diabetes patients who are mostly overnutrient, and may account for mild ketosis and erythropoiesis, which are associated with SGLT2i therapy^5,6^. However, it is important to note that SGLT2i consistently benefit HF patients irrespective of diabetes, established or risk of cardiovascular disease, or renal function^7,8^. Given the catabolic state in patients with HF, it is difficult to imagine that the favorable effects of SGLT2i on heart failure are due to the potentiation of nutrient-deprivation signaling in the energy-starved failing heart. Instead, an induction of nutrient-deprivation signaling by SGLT2i may be secondary to the improvement of pathophysiological conditions of HF such as adiposity and insulin resistance, among others. In this regard, it remains unknown whether SGLT2i induce the nutrient-deprivation signaling under the physiological conditions where organ systems are not bioenergetically challenged.

Skeletal muscle accounts for ~ 40% of body mass in non-obese subjects, and has ~ 30% of the resting metabolic rate^9^. Skeletal muscle possesses a robust capacity to remodel its metabolic machinery and can dynamically adapt to a wide range of physiological circumstances, such as exercise, fasting, and changes in hormonal profile, through structural, functional and metabolic plasticity^10^. For example, physical exercise, even a single bout of exercise, evokes transcriptional signaling pathways to coordinate the activity of multiple enzymatic cascades as a means to tightly couple gene expression with metabolic need and nutrient availability^11,12^. Intuitively, these forms of mechanistic plasticity of skeletal muscle are suitable to determine whether SGLT2i have the impact on energy metabolism in tissues under physiological circumstances.

The present study explores the effects of empagliflozin on the expression of the genes involved in the energy sensing, mitochondrial biogenesis, oxidative phosphorylation, mitochondrial respiration, and oxidative stress- resistance in skeletal muscle in healthy mice. In addition, we examined the effects of empagliflozin on these genes in the heart. Further, we analyzed the correlation between the expression of these genes and serum concentrations of metabolites.

## Results

### Effects of empagliflozin on the transcripts of the genes for key nutrient sensors

**Figure 1:**
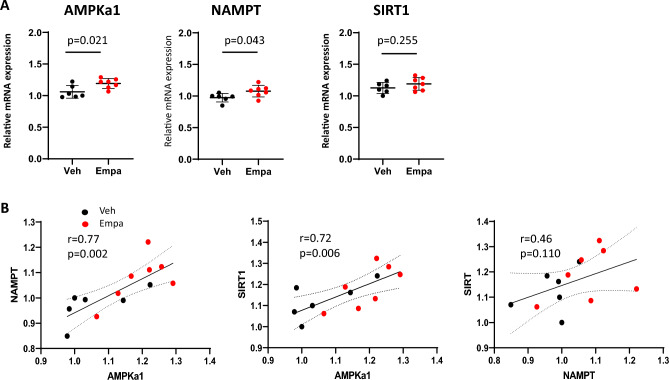
Effects of empagliflozin on energy sensing gene expression in quadriceps muscle. (**A**) Relative transcript levels of AMPKα1, NAMPT and SIRT1 in quadriceps muscle from vehicle-treated and empagliflozin-treated mice. Values represent mean ± SD (vehicle-treated, n = 6; empagliflozin-treated, n = 7). (**B**) Correlations between AMPKα1, NAMPT and SIRT1 gene expression. Relative transcript levels for AMPKα1, NAMPT and SIRT1 are shown in the *x*- and *y*-axis. A linear regression line, Pearson’s correlation coefficient and *p*-value are shown.

AMP-activated protein kinase (AMPK) is a prime nutrient sensor monitoring intracellular AMP levels and coordinates adaptive responses to fasting and exercise acutely through phosphorylation of metabolic enzymes, and chronically via transcriptional regulation^13^. Another metabolic sensor is NAD^+^-dependent deacetylase sirtuin SIRT1^14^. NAD^+^ biosynthetic enzyme nicotinamide phosphoribosyltransferase (NAMPT) is the nutrient-responsive gene that increases mitochondrial NAD^+^ levels^15^, and is required and sufficient for the activation of SIRT1^16^. To determine whether empagliflozin alters the transcription of these nutrient-sensing genes, we examined the transcripts for AMPKα1, NAMPT and SIRT1 in quadriceps muscle in mice treated with either vehicle or empagliflozin via intragastric administration of 10 mg/kg/day for 28 days under ad libitum feeding. The results of quantitative real-time PCR (qPCR) showed that both AMPKα1 and NAMPT transcripts significantly increased in empagliflozin-treated mice as compared with those in vehicle-treated mice (*p* = 0.021 and *p* = 0.043, respectively) (Fig. 0.1A). In addition, Pearson’s correlation analysis revealed that the transcripts levels of AMPKα1 and NAMPT strongly correlated each other (r = 0.77, *p* = 0.002). This result is consistent with the previous finding that the NAMPT gene transcription is induced by activated AMPK^17^. SIRT1 transcripts were comparable between the two groups (*p* = 0.255), but strongly correlated with AMPKα1 transcript (r = 0.72, *p* = 0.006) (Fig. 1B). These results suggest that empagliflozin activates the transcription of the genes required for cellular energy sensing in skeletal muscle.

### Effects of empagliflozin on the PGC-1α transcripts

**Figure 2:**
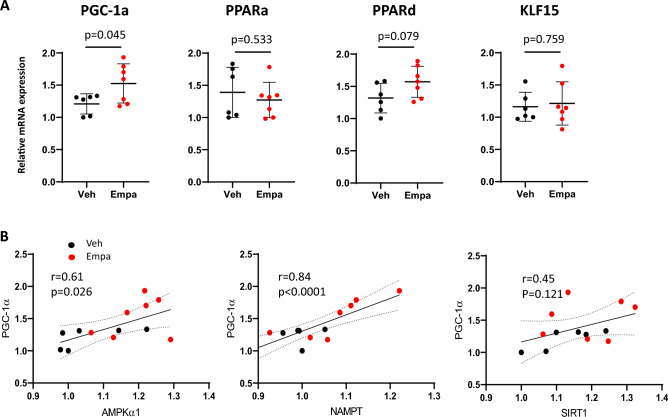
Effects of empagliflozin on the expression of transcription factors and coactivator in quadriceps muscle. (**A**) Relative transcript levels of PGC-1α, PPARα, PPARδ, and KLF15 in quadriceps muscle from vehicle-treated and empagliflozin-treated mice. Values represent mean ± SD (vehicle-treated, n = 6; empagliflozin-treated, n = 7). (**B**) Correlations between PGC-1α and energy sensing gene expression. Relative transcript levels for AMPKα1, NAMPT and SIRT1 are shown in the *y*-axis while those of PGC-1α are shown in the *x*-axis. A linear regression line, Pearson’s correlation coefficient and *p*-value are shown.

Peroxisome proliferator-activated receptor (PPAR)-γ coactivator-1α (PGC-1α) is a master regulator of metabolic programs for mitochondrial biogenesis and function^18^. Results of qPCR showed that PGC-1α transcripts significantly increased in empagliflozin-treated mice as compared with control mice (*p* = 0.045) in quadriceps muscle (Fig. 2A).

### Correlation between the nutrient sensors and the PGC-1 α transcripts

We next evaluated the coordinated regulation of AMPKα1, NAMPT, SIRT1 and PGC-1α expression. Pearson’s correlation analysis showed that the transcripts of the PGC-1α and AMPKα1 genes significantly correlated (r = 0.61, *p* = 0.026) (Fig. 2B). This observation is consistent with the previous data showing that the expression of PGC-1α gene is transcriptionally induced by AMPK activation^19^. PGC-1a transcripts were also highly correlated with NAMPT transcripts (r = 0.84, *p* < 0.0001) while the correlation between PGC-1α and SIRT transcripts was not statistically significant (r = 0.45, *p* = 0.121) (Fig. 2B). These results suggest that the expression of the AMPKα1, NAMPT and PGC-1α genes are closely linked together.

### Effects of empagliflozin on the PPARα, PPARδ and KLF15 transcripts

Much of the altered metabolic programming driven by PGC-1α is achieved by its binding to multiple transcription factors including nuclear receptors such as peroxisome proliferator-activated receptors (PPARs)^20^. PPARs are lipid-activated nuclear receptors involved in the regulation of mitochondrial enzymes involved in fatty acid metabolism and electron transport. We examined the effects of empagliflozin on the transcripts of PPARα and PPARδ. qPCR showed that PPARδ transcripts in skeletal muscle tended to be increased in empagliflozin-treated mice (*p* = 0.079) while PPARα transcripts were similar between the two groups (*p* = 0.533). The transcripts of the Krüppel-like factor 15 (KLF15), a critical regulator of lipid metabolism in skeletal muscle^21^, were also comparable between the two groups (Fig. 2A).

### Correlation between PGC-1α and mitochondrial gene transcripts

**Figure 3:**
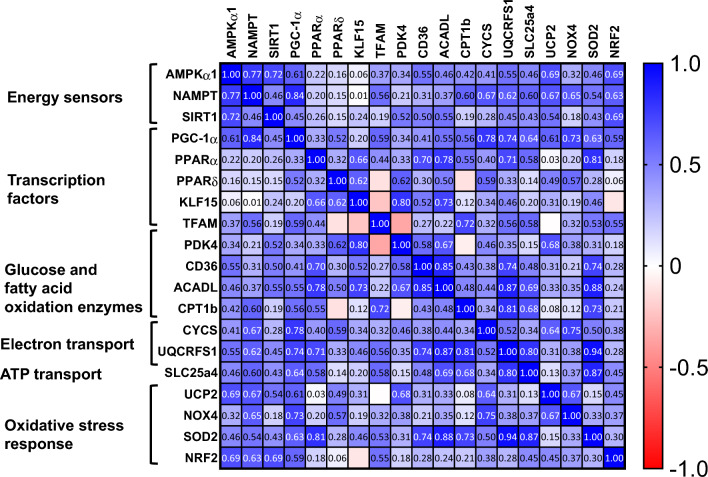
Pearson’s correlation coefficient matrix of multiple variables. Pearson’s correlation coefficient between the relative transcript levels of many genes involved in indicated biological processes are shown in matrix.

Skeletal muscle is a major user of fatty acids^10^. PPARs are fatty acid-activated transcription factors belonging to the nuclear receptor superfamily^22^. Consistent with the established role of PGC-1α-PPARδ complex in fatty acid catabolism^23^, Pearson’s correlation analysis showed that the transcripts of PGC-1α significantly correlated with those of many genes involved in fatty acid oxidation and oxidative phosphorylation such as acyl-CoA dehydrogenase long chain (ACADL), r = 0.55, *p* = 0.046; carnitine-palmitoyl transferase-1b (CPT1b), r = 0.56, *p* = 0.048; adenine nucleotide translocator 1 (SLC25a4), r = 0.64, *p* = 0.018; cytochrome c, somatic (CYCS), r = 0.78, *p* = 0.001; ubiquinol-cytochrome c reductase, Rieske Iron-Sulfur polypeptide 1 (UQCRFS1), r = 0.74, *p* = 0.004 (Fig. 3). In addition, expression of the mitochondrial transcription factor A (TFAM) gene, which is involved in mitochondrial biogenesis^24^, significantly correlated with that of PGC-1α (r = 0.59, *p* = 0.034). These results are consistent with the previous reports that PGC-1α controls the expression of genes related to mitochondrial biogenesis and function in skeletal muscle^25,26^.

### Effects of empagliflozin on transcripts of the genes for oxidative stress response

**Figure 4:**
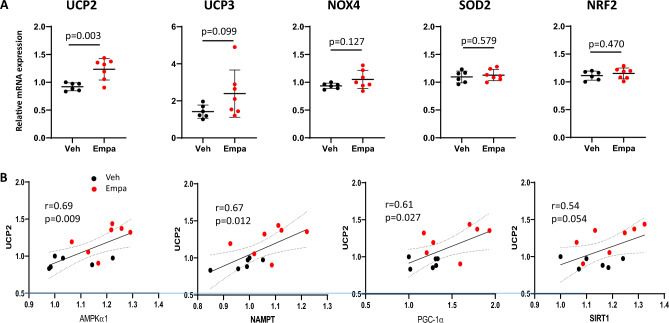
Effects of empagliflozin on the expression of oxidative stress response genes in quadriceps muscle. (**A**) Relative transcript levels of UCP2, UCP3, NOX4, SOD2, and NRF2 in quadriceps muscle from vehicle-treated and empagliflozin-treated mice. Values represent mean ± SD (vehicle-treated, n = 6; empagliflozin-treated, n = 7). (**B**) Correlations between UCP2 and the expression of the genes for nutrient sensors and PGC-1α. Relative transcript levels for AMPKα1, NAMPT, PGC-1α, and SIRT1 are shown in the *x*-axis while those of UCP2 are shown in the *y*-axis. A linear regression line, Pearson’s correlation coefficient and *p*-value are shown.

Uncoupling protein 2 and 3 (UCP2 and UCP3), the mitochondrial anion carriers that partially uncouple respiration from ATP synthesis, are expressed in skeletal muscle^27^. Fatty acids and superoxide activate UCP2 and UCP3 expression and the primary role of UCP2 and UCP3 has been suggested to be the protection against reactive oxygen species (ROS) produced during fatty acid oxidation^28^. qPCR showed that UCP2 transcripts were significantly increased (*p* = 0.003) in empagliflozin-treated group, although an increase in UCP3 transcripts did not reach statistical significance (*p* = 0.10) (Fig. 4A). Given a key role of PGC-1α in transcriptional induction of UCP2 gene expression ^29^, it is conceivable that the inducible expression of UCP2 by empagliflozin is at least partly due to the increased PGC-1α expression. Indeed, we found that the UCP2 transcripts significantly correlated with the AMPKα1 (r = 0.69, *p* = 0.009), NAMPT (r = 0.67, *p* = 0.012), and PGC-1α (r = 0.61, *p* = 0.002) transcripts.

Expression of other antioxidant defense genes including NADPH Oxidase 4 (NOX4), superoxide dismutase 2 (SOD2), and nuclear factor-erythroid-related factor (NRF2) did not significantly differ between the two groups. However, Pearson’s correlation analysis revealed that the expression of NOX4, SOD2 and NRF2 significantly correlated with that of PGC-1α (r = 0.73, *p* = 0.004; r = 0.63, *p* = 0.022; r = 0.59, *p* = 0.032, respectively) (Fig. 3). Additionally, the expression of NOX4 and NRF2 also significantly correlated with that of NAMPT (r = 0.65, *p* = 0.017 and r = 0.63, *p* = 0.020, respectively). These results suggest that the expression of energy-sensing genes is closely linked to that of the genes for oxidative stress response.

### Quantitative relationships between serum β-hydroxybutyrate (bOHB) and the transcripts of the nutrient-sensors and PGC-1α

**Figure 5:**
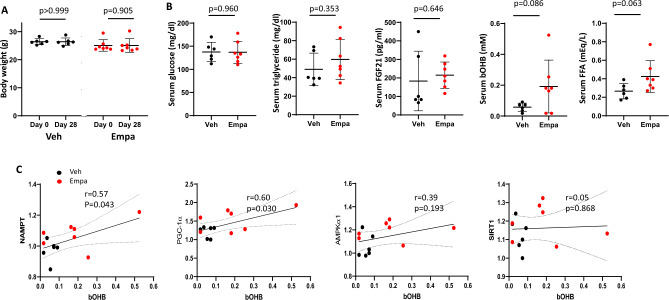
Body weight and serum parameters in vehicle- and empagliflozin-treated mice. (**A**) Body weight at day 0 and day 28 after vehicle or empagliflozin treatment. Values represent mean ± SD (vehicle, n = 6; empagliflozin, n = 7). (**B**) Serum concentrations of glucose, triglyceride, FGF21, bOHB, and FFA at day 28 after vehicle or empagliflozin treatment. Values represent mean ± SD (vehicle, n = 6; empagliflozin, n = 7). (**C**) Correlations between bOHB and the expression of the genes for nutrient sensors and PGC-1α. Relative transcript levels for NAMPT, PGC-1α, AMPKα1 and SIRT1 are shown in the *y*-axis while serum bOHB concentrations are shown in the *x*-axis. A linear regression line, Pearson’s correlation coefficient and *p*-value are shown.

To understand the upstream signals which potentially induce nutrient-sensing genes, we measured circulating metabolites. Serum glucose, triglyceride and FGF21 were comparable between the two groups, but serum bOHB and free fatty acid (FFA) were tended to be higher in empagliflozin-treated mice (*p* = 0.086, *p* = 0.063, respectively) (Fig. 5B). bOHB is increasingly appreciated to have cellular signaling functions in a variety of physiological contexts, including fasting and exercise^30^. Notably, both NAMPT and PGC-1α transcripts positively and significantly correlated with serum bOHB (r = 0.57, *p* = 0.043 and r = 0.60, *p* = 0.030, respectively) (Fig. 5C). Although it does not reach statistical significance, it may be important to note 3.4-fold increase in bOHB when comparing the means although it does not reach statistical significance. Taking these into consideration, an increased production of bOHB may be upstream of or associated with an increased expression of nutrient sensors and PGC-1α in empagliflozin-treated mice.

No correlation was found between serum FFA and the transcripts of the nutrient sensors and PGC-1α (Suppl. Fig.S1A). FGF21, a metabolic hormone mainly produced from liver, has been proposed to increase adipose tissue lipolysis and hepatic ketogenesis in response to fasting^31,32^. In the present study, serum FGF21 was comparable between the two groups, and no correlation was found between serum FGF21 and expression of nutrient sensors (Fig. 5, Suppl. Fig.S1B).

### Effects of empagliflozin on myogenic gene transcripts

Having the multi-dimensional role of energy metabolism in the regulation of muscle function, we next sought to determine the effects of empagliflozin on the myogenic gene expression (Suppl. Figure S1). The transcripts of the genes for master regulators of myogenesis, MyoD and MEF2 family (MEF2A and MEF2C), did not change between the two groups (data not sown). However, the gene encoding myocardin, a potent coactivator for serum response factor (SRF) that binds to the sequence [CC(A/T)6GG] termed a CArG box or serum response element (SRE)^33^, increased significantly in empagliflozin-treated mice (Suppl. Fig. S2A). In addition, Pearson’s correlation analysis revealed that the myocardin transcripts highly correlated with the AMPKα1 (r = 0.77, *p* = 0.002), NAMPT (r = 0.81, *p* = 0.001) and SIRT1 (r = 0.67, *p* = 0.011) transcripts (Suppl. Fig. S2B). While the myocardin expression has been reported to be restricted to cardiac and smooth muscle, the myocardin-related transcription factors MRTF-A and MRTF-B genes are broadly expressed^34^. But neither MRTF-A nor MRTF-B gene expression changed in empagliflozin-treated mice (data not shown). These results suggest that empagliflozin transcriptionally induces the myocardin expression in association with the expression of the nutrient sensors.

### Effects of empagliflozin on transcripts of nutrient sensors and PGC-1α in the heart

**Figure 6:**
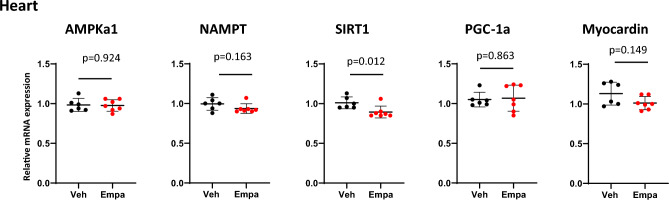
Effects of empagliflozin on the expression of the genes for nutrient sensors, PGC-1α, and myocardin in the heart. Relative transcript levels of AMPK α 1, NAMPT, SIRT1, PGC-1 α, and myocardin genes in the heart from vehicle-treated and empagliflozin-treated mice. Values represent mean ± SD (vehicle-treated, n = 6; empagliflozin-treated, n = 7).

Finally, we examined whether empagliflozin has a similar effect on the transcriptional program in the heart. qPCR analysis showed that empagliflozin did not affect the expression of the AMPKα1, NAMPT, and PGC-1α genes and that it significantly decreased the SIRT1 expression (*p* = 0.012) (Fig. 6). The myocardin expression, which is abundant in the heart, was not affected. These results suggest that empagliflozin has little, if any, effect on the transcriptional program related to metabolism in the heart, and thus, the heart is less susceptible to empagliflozin than skeletal muscle in a healthy state.

## Discussion

We report that empagliflozin significantly and coordinately induces the expression of the genes for nutrient sensors, AMPKα1 and NAMPT, and their downstream target PGC-1α in skeletal muscle in normal mice under ad libitum feeding. Increasing numbers of studies showed that SGLT2i increased the activation of these mediators of mitochondrial metabolism in a variety of experimental models such as diabetic mice, high-fat diet-induced obese mice, Dahl-sensitive rats, cultured cardiomyocytes exposed to lipopolysaccharide, doxorubicin or angiotensin II (see reviews^4,35^). In addition, it is well established that AMPKα1, NAMPT, and PGC-1α form a molecular network that plays an important role in cellular energy homeostasis in skeletal muscle^36^. Therefore, the coordinated induction of these genes is not surprising. However, because our mouse model does not have confounding metabolic abnormalities, this study provides novel evidence that empagliflozin activates the transcriptional programs for energy homeostasis as a physiological response in the skeletal muscle, not a secondary response to the alleviation of underlying metabolic derangements.

Which factors are responsible for the activation of nutrient-sensing genes by SGLT2i in skeletal muscle? SGLT2i cause weight loss, although substantially less than expected from the urinary glucose excretion^37^. In this study, the change in body weight after empagliflozin treatment for 28 days was almost identical with that in vehicle-treated controls, suggesting that empagliflozin-treated mice were likely to compensate for glycosuria-induced caloric loss by increasing food intake (hyperphagia), as reported by Devenny et al. who used dapagliflozin-treated diet-induced obese (DIO) rat model^38^. Therefore, the activation of nutrient-sensing genes by SGLT2i in skeletal muscle is weight independent.

**Figure 7:**
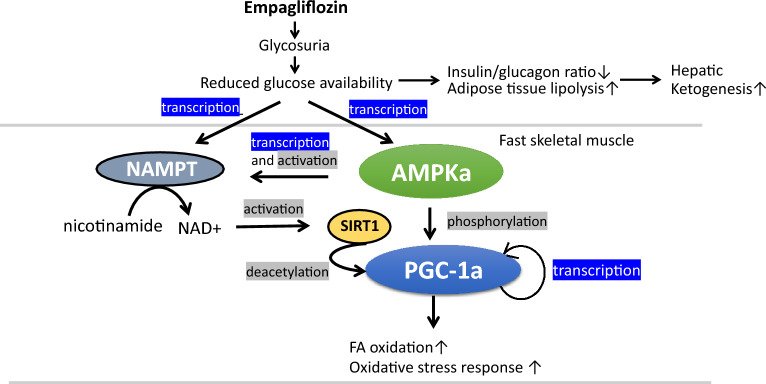
Schematic model of effects of empagliflozin on skeletal muscle metabolism. Our own findings in the present study are indicated by blue rectangles, and established mechanisms by many prior studies are indicated by gray rectangles. Empagliflozin-induced glycosuria renders glycolytic fast muscle nutrient-deprived, and increases the transcription of the AMPKα1 and NAMPT genes. PGC-1α is expected to be activated by phosphorylation and deacetylation via AMPKα and SIRT1, respectively. PGC-1α activates its own promoter as well as its downstream target genes, which are involved in FA oxidation and oxidative stress response. Empagliflozin-induced glycosuria alters systemic hormonal environment and promotes adipose tissue lipolysis and subsequent hepatic ketogenesis.

We reason that in empagliflozin-treated mice, urinary caloric loss is compensated by overeating, but glucose loss is only partly compensated by carbohydrate in the standard chow, and therefore, glucose available to the energy-demanding organs including skeletal muscle is relatively reduced compared with control mice. It is well documented that glucose restriction activates AMPK independently of any changes in intracellular AMP/ATP and ADP/ATP ratios in mouse embryo fibroblasts (MEFs)^39,40^. Likewise, glucose restriction induces AMPK activity and NAMPT transcription even under the conditions where fatty acid oxidation is increased in skeletal myoblasts^25^. Fast skeletal muscle including quadriceps preferentially metabolize glucose and is expected to be more susceptible to glucose restriction compared with slow skeletal muscle. In fact, Otsuka et al. demonstrated that canagliflozin tended to increase AMPK phosphorylation in fast skeletal muscle preferentially over slow skeletal muscle in nondiabetic mice^41^. Taking these into consideration, we assume that a reduced glucose availability may underlie the empagliflozin-induced activation of nutrient-sensing genes in quadriceps muscle (Fig. 7).

Growing numbers of evidence indicate that bOHB acts as an endogenous inhibitor of class 1 histone deacetylases (HDACs) activation, and increases the transcription of the genes encoding oxidative stress resistance genes^30^. Consistently, serum bOHB correlated with AMPKα1, NAMPT, PGC-1α (Fig. 4B), and UCP2 expression (r = 0.698, *p* = 0.008). However, a ketogenic diet did not induce PGC-1α expression despite the induction of AMPK activity in the liver^42^. Further, we did not find little, if any, effect of bOHB on the expression of the nutrient-sensing genes in differentiated C2C12 cells (Suppl. Fig. S3). These data argue against the notion that bOHB plays a role as a physiologically relevant mediator of the effects of empagliflozin on nutrient-sensing gene induction in skeletal muscle. Instead, both ketogenesis and nutrient-sensing gene expression may be induced by common factor(s).

Among many factors which facilitate hepatic ketogenesis by SGLT2i, primary determinant may be low glucose^5,43,44^.The observed association between bOHB and nutrient-sensing gene expression in empagliflozin-treated mice may be interpreted such that low glucose per se induces both ketogenesis in the liver and nutrient-sensing gene expression in skeletal muscle independently. Previous studies showed that PPARα-FGF21 axis promotes fasting-induced ketogenesis^31,32^, and canagliflozin increases fasting serum FGF21 which facilitates lipolysis in adipose tissue, and resultantly, increases bOHB production in diet-induced obese mice^45^. However, we herewith find that empagliflozin has no effect on serum FGF21, and thus, FGF21 is unlikely to be a relevant mediator in our model.

Consistent with the well-defined role of PGC-1α-PPARs axis in the transcriptional regulation of the genes involved in fatty acid metabolism^46^, Pearson’s correlation analysis revealed that the expression of the genes related to mitochondrial metabolisms such as fatty acid transport, its oxidation, electron transport, and mitochondrial biogenesis, significantly correlated with the expression of either PPARα or PPARδ. Nevertheless, the difference in the expression levels of these genes between vehicle- and empagliflozin-treated mice was not statistically significant. The lack of significant activation of the mitochondrial genes despite a significant increase in AMPKα1 and PGC-1α expression is likely due to the mouse model we used. To avoid cofounding factors including energetic stresses, we used healthy mice under ad libitum feeding. The significant correlation between nutrient-sensing genes and many genes related to mitochondrial metabolism led us to speculate that the activation of nutrient-sensing genes could potentially translate to the upregulation of mitochondrial oxidation and anti-oxidative defense gene expression under the condition where muscle is subjected to profound energetic deficiency such as long-term fasting and endurance exercise.

In this context, it is worth noting that empagliflozin significantly induces the expression of UCP2 in parallel with AMPKα, NAMPT, and PGC-1α expression. This observation is consistent with the previous reports showing that the UCP2 gene is AMPK target in skeletal muscle^47^. UCP2 is a mitochondrial protein that regulates mild uncoupling and activates in response to subtle mitochondrial ROS produced by the protonmotive force set up across the inner membrane by electron transport^28,48^. Therefore, empagliflozin-induced UCP2 expression may be a transcriptional signature of the increased fatty acid oxidation and oxidative phosphorylation in skeletal muscle.

The clinical relevance of this study is two-fold. First, our data support the notion that SGLT2i induce the metabolic state similar to fasting, which could reverse the abnormality of nutrient-related signaling pathways in the heart and kidney^4^. However, our findings that empagliflozin had no effect on the nutrient-sensing signaling in normal heart suggest that the activation of nutrient-deprivation signaling postulated in the failing heart may be a consequence of the amelioration of the underlying pathophysiology such as mitochondrial dysfunction, oxidative stress, and lipotoxicity.

Secondly, our findings may explain the favorable clinical effects of SGLT2i on exercise performance in patients with HF as assessed by patient-reported symptoms and other measures of physical limitations and exercise function^49–51^. Exercise intolerance is highly prevalent in patients with HF, and a fundamental component of the condition of this disease^52^. A number of reports using biopsy specimens and in vivo measurements with phosphorus-31 magnetic resonance spectroscopy (MRS) demonstrated that percent of type I (oxidative) muscle fibers relative to type II (glycolytic) muscle fibers is reduced, and mitochondrial oxidative metabolism is impaired in skeletal muscle in patients with HF irrespective of left ventricular ejection fraction^53–55^. Type II muscle fibers, as in quadriceps muscle, can undergo switching to fatigue-resistant type I fibers in response to endurance exercise through the mechanisms involving AMPK and PGC-1α activation^19,47^. Taken together, our findings raise the intriguing possibility that clinical benefits of SGLT2i are partly attributed to the activation of signaling pathways involving AMPKα1, NAMPT, and PGC-1α in skeletal muscle which have been advocated as the molecular mediators of muscle adaptation to endurance exercise^12,56,57^. To our knowledge, no prior studies have explored the effects of SGLT2i on the oxidative metabolism in the skeletal muscle in vivo using phosphorus-31 MRS methodology. Further studies should be warranted to test this hypothesis.

Of note, empagliflozin had virtually no effects on the expression of genes for nutrient sensing network in the heart. But we should be aware that many cardiovascular medications exert their effects differently depending upon the underlying pathophysiological state. For example, statins prevent atherosclerotic events more strongly in patients with higher levels of plasma inflammatory markers (e.g., C-reactive protein) at baseline^58^, and benefit of treatment with renin-angiotensin system (RAS) inhibitors or sacubitril-valsartan is larger in HF patients with reduced ejection fraction (HFrEF) than in those with preserved ejection fraction (HFpEF)^59^. Thus, we can envisage that empagliflozin primarily exerts its rescue effects on metabolism of failing heart, particularly in HFrEF, in which energy production is generally compromised^60^. However, it is important to emphasize that the results in this study may help us understand the mechanisms by which empagliflozin consistently improve exercise tolerance in patients with HFpEF, in which a consensus on cardiac metabolic changes has yet been identified^61^.

### Limitation of study

Our study has several limitations. First, this study solely used qPCR and did not examine the protein expression levels of nutrient-sensing genes in skeletal muscle. Neither phosphorylation nor deacetylation activities of AMPK and SIRT1, respectively, were not examined. Further, the transactivation function of PGC-1α remains unanswered. As a result, we cannot confirm that components of nutrient-sensing network are activated at protein levels by empagliflozin. Obviously, however, the advantage of qPCR is to be able to reliably quantitate the expression of the genes that do not exhibit large change in transcription. Secondly, serum glucose levels were measured only once before sacrifice. Thus, we cannot conclusively state that empagliflozin lowered glucose levels. Nevertheless, because our mice model maintained a normal feeding and locomotor activity during the nighttime, and empagliflozin was administered at daytime, we expect that glycosuria-induced glucose loss is not being compensated by eating during the daytime when frequency of food intake is much less than that in night-time. Precise monitoring is required to detect the difference in serum glucose levels between the two groups when studying mice under ad libitum feeding. Thirdly, numbers of mice examined may be too small to substantiate the statistical analysis. Thus, we performed the correlation analysis of vehicle-treated and empagliflozin-treated groups, separately. Interestingly, Pearson’s correlation coefficients (r) among nutrient sensing network gene transcripts (i.e., AMPKα1, NAMPT, SIRT1, and PGC-1α) remained larger than 0.60 in vehicle-treated group (Suppl. Fig. S4). These results suggest that there intrinsically exist positive correlations between the expression of each of nutrient sensing network genes in skeletal muscle. However, it is obvious that validation study using larger numbers of mice should be necessary. Fourthly, we did not explore the role of AMPK-NAMPT-PGC-1α pathway in the regulation of mitochondrial metabolism in the skeletal muscle. However, we investigated the effects of PGC-1α knockdown on mitochondria-related gene expression in vitro using C2C12 cells. Results showed that PGC-1α knockdown clearly reduced the expression of a broad array of genes involved in mitochondrial metabolism. Of a particular interest, PGC-1α knockdown substantially reduced the expression of AMPKα1, NAMPT, PPARα, and SIRT1 (Suppl. Fig. S5), suggesting that there seem to be feed-forward relationship among PGC-1α AMPKα1, NAMPT and SIRT1 gene expression. These results conform to the established notion that PGC-1α gene plays a key role in mitochondrial metabolism^56^ and suggest the novel possibility that PGC-1α is required for the expression of nutrient sensing genes. Lastly, although we highlighted the positive effects on fast skeletal muscle, the effects on slow skeletal muscle remains speculative.

## Conclusions

This study demonstrates that empagliflozin significantly and coordinately induces the expression of genes critical for low nutrient-sensing programs represented by AMPK-NAMPT-PGC-1α network in skeletal muscle, even in the absence of caloric loss. Notably, activation of these genes in metabolically healthy mice suggest that empagliflozin enhances the intrinsic capability of skeletal muscle to adapt to changes in nutrient availability. These findings may open new possibility of expanding the targets of SGLT2i to skeletal muscle.

## Materials and methods

An expanded methods section can be found in Supplementary material.

### Animal care

C57BL/6 strain (WT) mice were purchased from CLEA Japan Inc. An 8–10-week-old mice were used. The Institutional Animal Care and Use Committee (Gunma University Graduate School of Medicine) approved all studies. Animal experiments conformed to the NIH guidelines (Guide for the Care and Use of Laboratory Animals). All authors complied with the ARRIVE (Animal Research: Reporting of In Vivo Experiments) guidelines. The mice were housed in a temperature-controlled room (20–26 ℃) with a 12 h light/12 h dark cycle and given unrestricted access to water and standard chow (CE-2, Clea Japan, Inc.): 12% of energy from fat, 29% of energy from protein, and 59% of energy from carbohydrate. Empagliflozin was suspended with 0.5% methylcellulose solution (Wako Inc.) and intragastrically administered to mice at a dose of 10 mg/kg. The same amount of methylcellulose solution was administered to control mice. Euthanization of mice was performed under 2% isoflurane anesthesia by intracardiac injection of 200 ml 5% potassium chloride to induce cardiac arrest.

### RNA isolation and quantitative real-time reverse transcription (qPCR)

Total RNA was extracted from the mouse quadriceps muscle using ISOGEN regent (Takara Bio) according to the manufacturer’s protocol. One microgram of RNA was used for reverse transcription with the ReverTra Ace RT-PCR Kit (TOYOBO) and qPCR analysis was performed using the THUNDERBIRD SYBR qPCR Mix (TOYOBO) according to the manufacturers’ protocols. QPCR was carried out using a StepOne real time PCR system (Applied Biosystems). Delta Ct values were calculated using Nuclear single-copy housekeeping gene 36B4 as a reference gene. All primer sequences are shown in Supplementary Table 1.

### Statistical analysis

All statistical analyses were performed using Prism 8 (GraphPad Software). For comparisons of 2 intervention groups (vehicle- versus empagliflozin-treated mice) with 1 variable (n = 5, and n = 6, respectively), an F-test was applied to determine if variance between groups differed significantly. If variance did not differ significantly, a 2-tailed unpaired Student’s *t*-test was applied. If F-test results showed significant difference in variance, a 2-tiled unpaired t-test with Mann Whitney U test was applied. Paired Student’s *t*-tests for body weight before and after intervention which have variances were used to calculate the *p* values. All continuous variables are presented as the mean ± standard deviation (SD). Two-sided *p*-value < 0.05 was considered statistically significant.

### Supplementary Information

Supplementary Information.

## Data Availability

All the data supporting the findings of this study are available from the corresponding author on reasonable request.
