# Supplementary material for: Empagliflozin induces the transcriptional program for nutrient homeostasis in skeletal muscle in normal mice
Source: Sci Rep. 2023 Oct 21;13:18025. doi: 10.1038/s41598-023-45390-y (PMC10590450; doi:10.1038/s41598-023-45390-y)
Supplement: Supplementary file 1 — Supplementary Information. [file 41598_2023_45390_MOESM1_ESM.pdf]

Supplementary Material

Empagliflozin induces the transcriptional program for nutrient homeostasis in skeletal muscle in normal mice

Ryo Kawakami[1]†, Hiroki Matsui[2]†, Miki Matsui[1], Tatsuya Iso[1],

Tomoyuki Yokoyama[2], Hideki Ishii[1], and Masahiko Kurabayashi[1]*

[1]Department of Cardiovascular Medicine, Gunma University Graduate School of Medicine, Maebashi, Gunma, Japan; [2]Department of Laboratory Sciences, Gunma University Graduate School of Health Sciences, Maebashi, Gunma, Japan.

†These authors contributed equally to this work

Short title: Effects of Empagliflozin on Skeletal Muscle

***Address for Correspondence**

Masahiko Kurabayashi, MD, PhD

Department of Cardiovascular Medicine, Gunma University Graduate School of Medicine,

3-39-15 Showa-machi, Maebashi, Gunma 371-8511, Japan

Tel.: +81-27-220-8140; Fax: +81-27-220-8150; E-mail address: mkuraba@gunma-u.ac.jp

1. Methods

Cell culture

C2C12 cells were grown in Dulbecco's modified Eagle medium (DMEM) supplemented with 20% fetal bovine serum (growth medium, GM). For differentiation into multinucleate myotubes, nearly confluent cultures (60%–70% confluency) were switched to nutrient poor medium (DMEM supplemented with 2% horse serum (differentiation medium, DM) [1]. Extensive myoblast fusion was obtained after 24 to 36 h. 96 h after switching to DM, cells were cultured in serum free DMEM containing glucose of 5.6mM (100mg/dL) for 24 h and stimulated with bOHB of indicated concentrations for 24 h before harvest.

Reference

1 Sartorelli, V. *et al.* Acetylation of MyoD directed by PCAF is necessary for the execution of the muscle program. *Mol Cell* **4**, 725-734 (1999).

2. Results

Quantitative relationships between serum FFA or FGF21 and the transcripts of the nutrient sensors and PGC-1α.

Pearson's correlation analysis showed that neither FFA nor FGF21 correlated the transcript levels of the nutrient sensors, AMPKα1 and NAMPT, and coactivator PGC-1α (Suppl.Fig.S1).

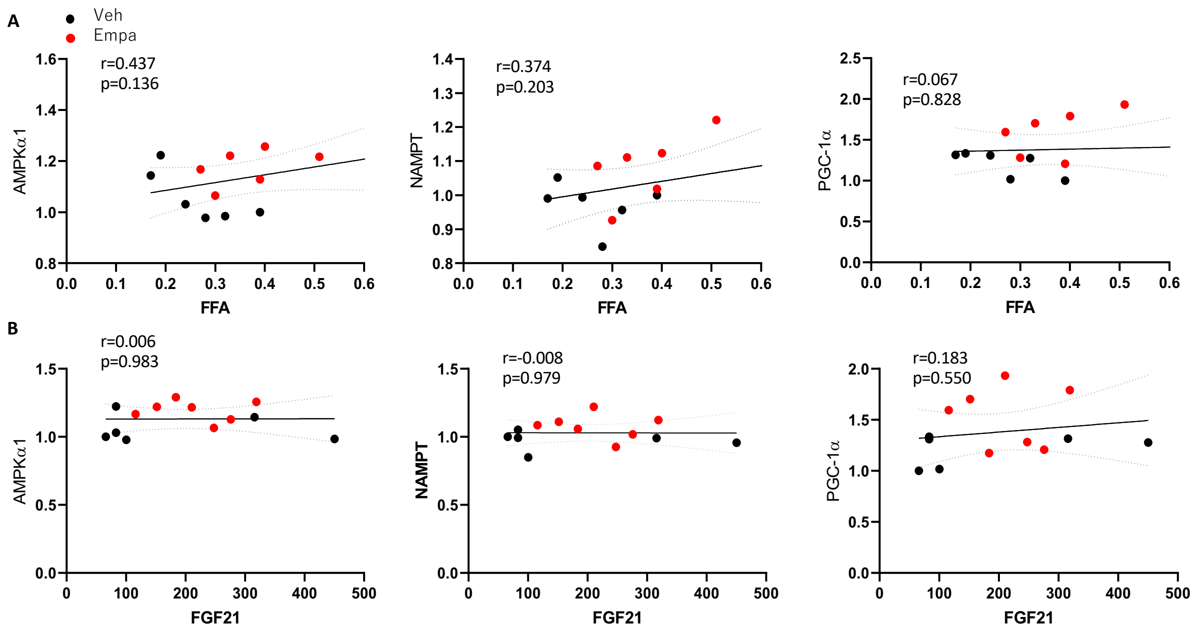

Suppl. Fig. S1. Correlations between serum FFA or FGF21 and the transcripts of the nutrient sensors and PGC-1α. Relative transcript levels for the AMPKα1, NAMPT, and PGC-1α genes are shown in the *y*-

axis while serum FFA (A) or FGF21 (B) concentrations are shown in the *x*-axis. A linear regression line, Pearson's correlation coefficient and p-value are shown.

Effects of empagliflozin on myogenic gene expression.

We examined the effects of empagliflozin on the transcript levels of the genes encoding transcription factors or coactivators, including, MyoD, MEF2A, MEF2C, myocardin, myocardin-related transcription factor (MRTF-A, MRTF-B) in skeletal muscle. Among the genes tested, the myocardin transcripts significantly increased in empagliflozin-treated mice compared with that in vehicle-treated mice (p=0.030) (Suppl.Fig.S2A). In addition, the transcripts for the myocardin gene significantly correlated with those for nutrient sensors, PGC-1α, and mitochondrial metabolism genes (Suppl.Fig.S2B).

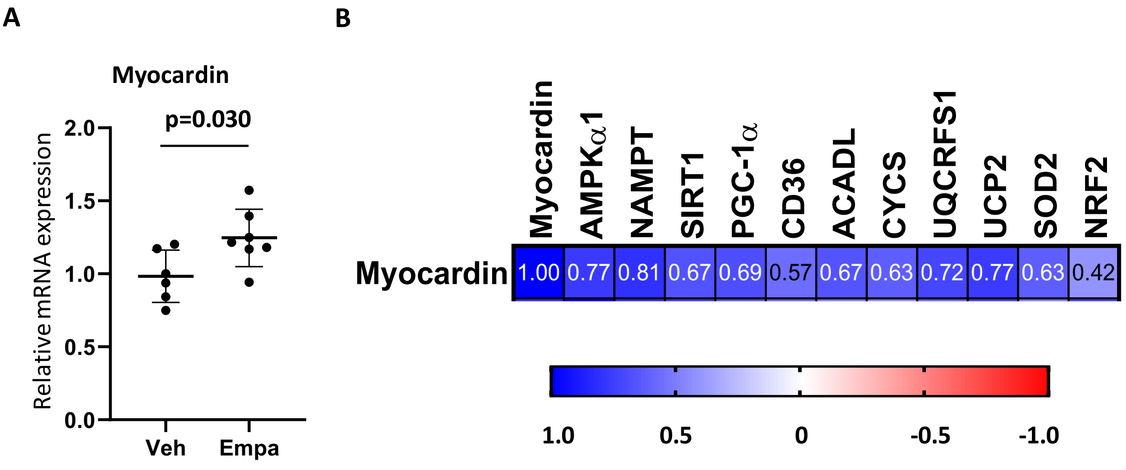

__Suppl. Fig. S2.__ **Effects of empagliflozin on myocardin gene expression in skeletal muscle.**

(A) Relative transcript levels for the myocardin gene in quadriceps muscle from vehicle-treated and empagliflozin-treated mice. Values represent mean ± SD (vehicle-treated, n=6; empagliflozin-treated, n=7).

(B) Pearson's correlation coefficient between the relative transcript levels for the myocardin and many genes involved in multiple biological processes.

Effects of bOHB on the transcripts of the nutrient-sensing genes and PGC-1α gene in differentiated C2C12 cells.

Effects of bOHB on the transcript levels for the AMPKα1, NAMT, SIRT1, and PGC-1α genes in the differentiated C2C12 cells were examined by qPCR. Results showed that bOHB tended to decrease the transcript levels for each of these genes in a dose-dependent manner in the single experiment (Suppl.Fig.S3).

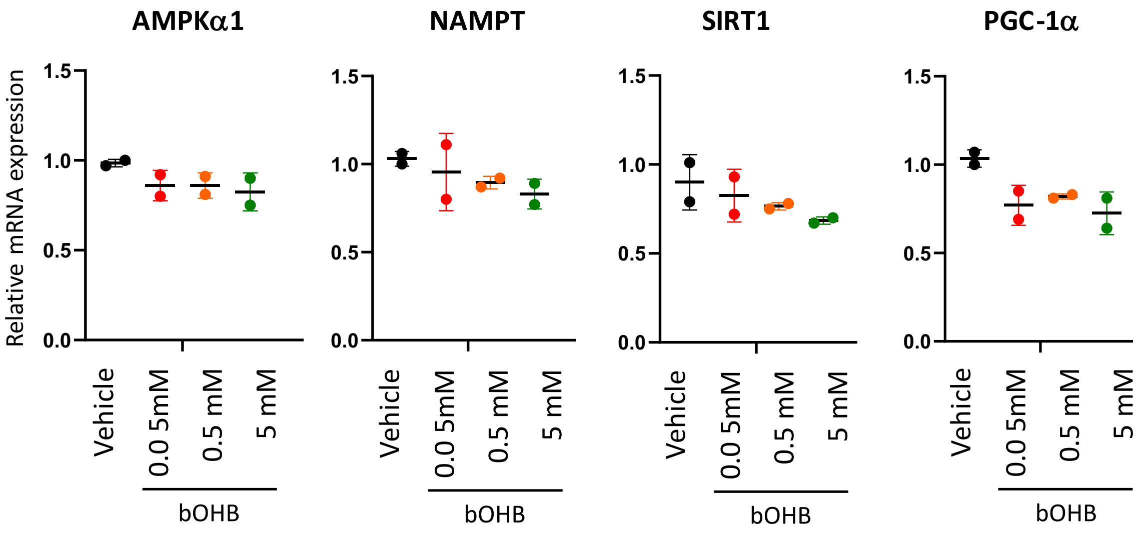

<u>Suppl. Fig. S3.</u> Effects of bOHB on the expression of the genes for nutrient sensors and PGC-1α in differentiated C2C12 cells.

Differentiated C2C12 cells were stimulated with bOHB of indicated concentrations for 24 h. Transcript levels for each of the AMPKα1, NAMPT, SIRT1 and PGC-1α genes were expressed as relative levels of vehicle-treated cells. The transcripts were normalized to those of 36B4 gene.

Separate analyses of vehicle- and empagliflozin-treated group.

We compare Pearson's correlation coefficient (r) between individual group and combined group. Results showed that Pearson's correlation coefficients (r) among three nutrient sensing gene transcripts (i.e., AMPKα1, NAMPT, and SIRT1) are larger than 0.60 almost consistently in vehicle-treated group (Suppl. Fig. S4).

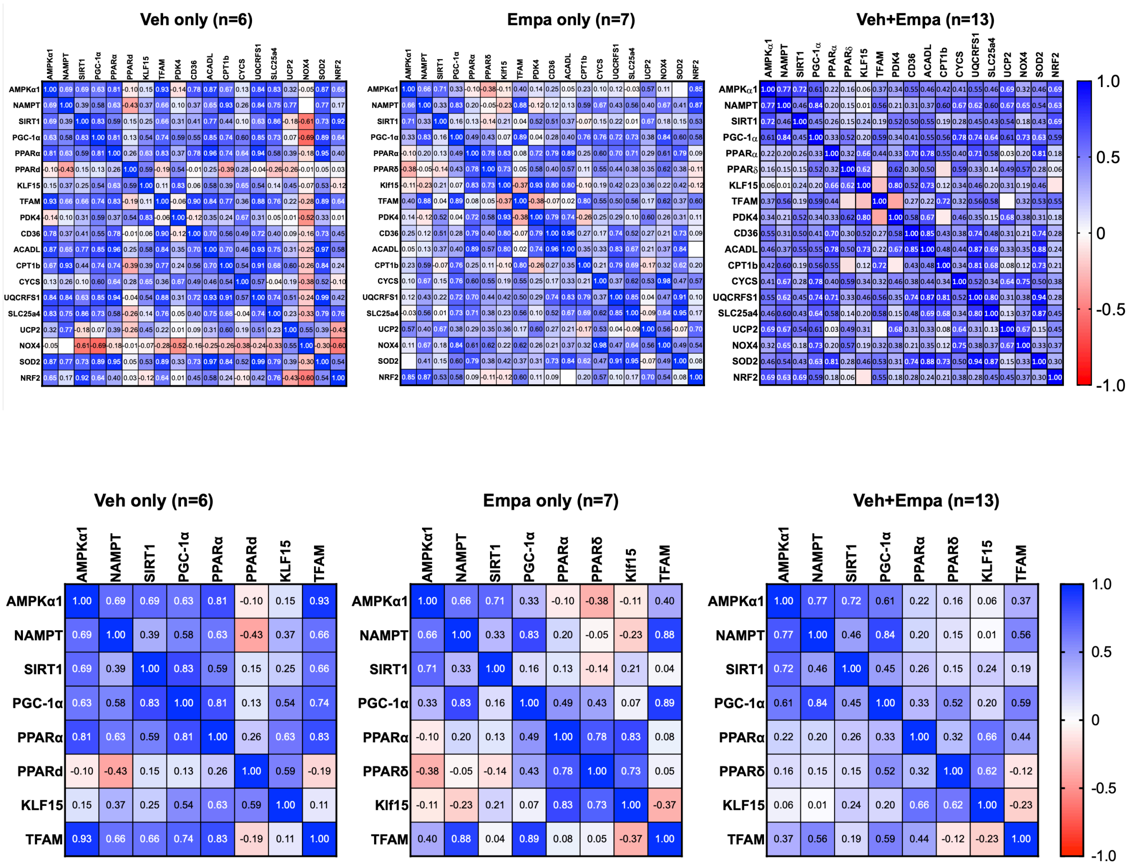

<u>Suppl. Fig. S4.</u> Pearson's correlation coefficient in vehicle-, empagliflozin-treated group and combined groups.

Pearson's correlation efficient (r) between transcripts of 19 genes (upper panel) and 8 genes (lower panel) relevant to nutrient sensing enzymes, transcription factors and cofactors genes is shown.

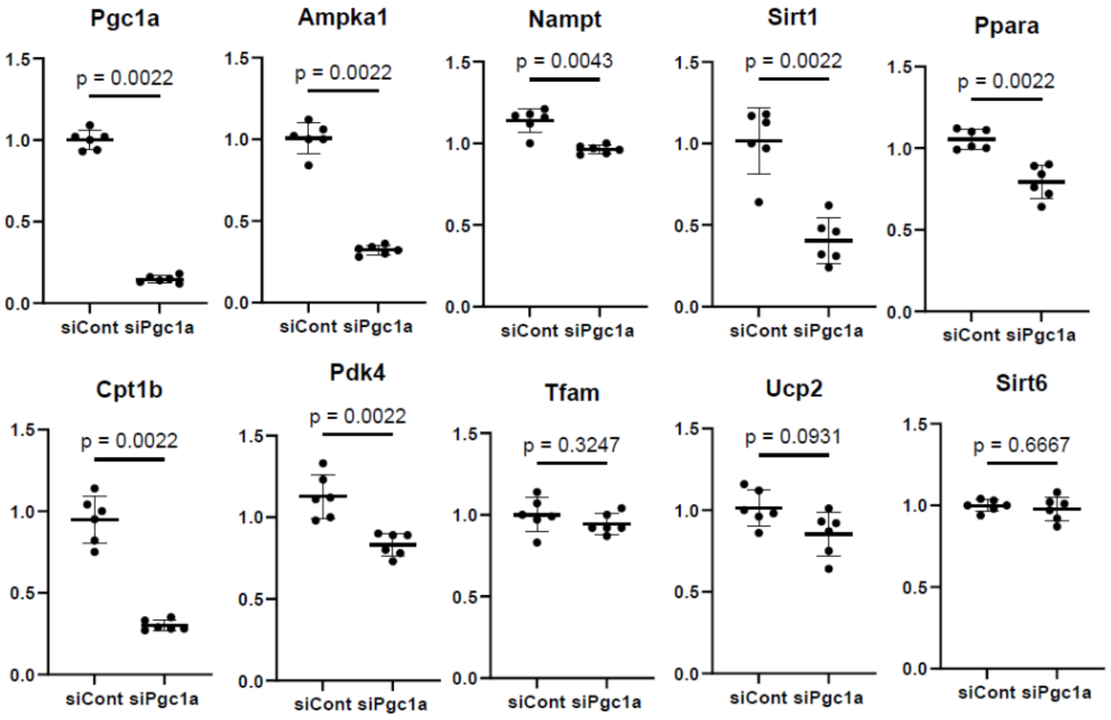

<u>Suppl. Fig. S5.</u> Effects of PGC-1α knockdown on the expression of genes for mitochondria metabolism in C2C12 cells.

Mouse skeletal muscle C2C12 cells transduced with siRNA for PGC-1α were subjected to qPCR analysis of the indicated genes. PGC-1α knockdown broadly but selectively reduced the expression of the genes involved in nutrient sensing and oxidative phosphorylation.

Suppl. Table 1

List of primer sets used for qPCR

Primers set	Forward	Reverse
**AMPKα1**	5'-tcatgtggctgggtgtgtaaagata-3'	5'-ggcattccattcatcatgtcagag-3'
**NAMPT**	5'-tgtcctgcatgggtataggcatag-3'	5'-agccggtggaattcacacaa-3'
**SIRT1**	5'-gcagacgtggtaatgtccaaacag-3	5'-acatcttggcagtatttgtggtgaa-3'
**PGC-1α**	5'-ttgacagctgcattcatttatcacc-3'	5'-aacacttgagcaagcattcgaca-3'
**PPARα**	5'-ctcagggtaccactacggagttcac-3'	5'-tgaatcttgcagctccgatcac-3'
**PPARδ**	5'-cagctcacaggcagagttgcta-3'	5'-caagccacttgaagcagcaga-3'
**Myocardin**	5'-ggaagccatcctgtgcttgag-3'	5'-tcctgggaattgattggtctgtc-3'
**KLF15**	5'-aagtttcccaagaacccagca-3'	5'-gacaactcatctgagcgggaaa-3'
**TFAM**	5'-gcagcaggcactacagcgatac-3'	5'-ctgagctccgagtccttgaacac-3'
**PDK4**	5'-gggtctcaatagtgtcacctgtgta-3'	5'-aagtgggcctgggcatttag-3'
**CD36**	5'-tgctggagctgttattggtg-3'	5'-tctttgatgtgcaaaaccca-3'
**ACADL**	5'-acttgggaagagcaagcgta-3'	5'-ttccgttttccaccaaaaag-3'
**CPT1b**	5'-gtcgcttcttcaaggtctgg-3'	5'-aagaaagcagcacgttcgat-3'
**CYCS**	5'-cattaccctggtgtggctttca-3'	5'-atgccataaatacagcagccattag-3'
**UQCRFS1**	5'-gtgaagcgacccttcctgtg-3'	5'-gcctcactgctttctttagaagatt-3'
**SLC25A4**	5'-tctgtccagggcatcatcatctac-3'	5'-ctgggcaatcatccagctca-3'
**UCP2**	5'-actctgccttgggccagta-3'	5'-gctgctcataggtgacaaacatc-3'
**UCP3**	5'-ctctgcactgtatgctgaagatg-3'	5'-cacgttccaagctcccaga-3'
**SOD2**	5'-gagaatctcagtgctcactcgtgtc-3'	5'-ggaaccctaaatgctgccagtc-3'
**NRF2**	5'-tccgctgccatcagtcagtc-3'	5'-attgtgccttcagcgtgcttc-3'
**36B4**	5'- atccctgacgcaccgccgtga -3'	5'- tgcatctgcttggagcccacgtt -3'
